# Clinical and Biochemical Characteristics of COVID-19-Associated Acute Kidney Injury (COVAKI): A Proof-of-Concept Case-Control Study

**DOI:** 10.7759/cureus.57763

**Published:** 2024-04-07

**Authors:** Girish V Kumthekar, Manasi S Nagarkar, Veena Purandare, Sharvari Shukla, Rajiv Yeravdekar

**Affiliations:** 1 Nephrology, Symbiosis Medical College for Women, Symbiosis University Hospital and Research Centre, Symbiosis International University (SIU), Pune, IND; 2 Medicine, Symbiosis Medical College for Women, Symbiosis University Hospital and Research Centre, Symbiosis International University (SIU), Pune, IND; 3 Internal Medicine, Symbiosis Medical College for Women, Symbiosis University Hospital and Research Centre, Symbiosis International University (SIU), Pune, IND; 4 Biostatistics and Epidemiology, Symbiosis Statistical Institute, Pune, IND; 5 Obstetrics and Gynaecology, Faculty of Medical and Health Sciences, Symbiosis International University (SIU), Pune, IND

**Keywords:** hospital-acquired acute kidney injury (haaki), : acute kidney injury, covid-19, renal replacement therapy (rrt), covaki

## Abstract

Introduction

Acute kidney injury (AKI) develops in 20-70% of patients with COVID-19. AKI is a syndromic diagnosis with multiple causes and outcomes. This cross-sectional study explored different outcomes of AKI in patients admitted with COVID-19.

Material and methods

It was a cross-sectional and descriptive study carried out in a tertiary care teaching hospital in Western Maharashtra for two months (May to June 2020). We collected clinical and laboratory data of 456 inpatients admitted with COVID-19 over two consecutive months. We excluded patients already on dialysis upon arrival at the hospital. It predominantly consists of patients who developed AKI during their stay in the hospital.

Result

C-reactive protein (CRP) was elevated in patients with COVID-19 associated with AKI (COVAKI) (78.87) but was statistically significant (p<0.003). Ferritin was elevated significantly (1619.19) in patients with AKI (p<0.0001). Similarly, higher levels of D-dimer (426.35) and lower serum albumin (1.86) were associated with COVAKI (p<0.0001). The average ICU stay was six days for patients with AKI and 0.37 days for patients without AKI. Days on the ventilator were 3.3 days for patients with AKI and 0.11 days for non-AKI patients. Out of a total 12 deaths of COVID-19 patients over these two months, nine had AKI. This made the association statistically significant (p<0.0001).

Conclusion

The phenotype COVAKI was associated with higher inflammatory markers, prolonged hospital stay, days spent on a ventilator, and higher oxygen requirement translating into higher mortality compared to those without COVAKI. We found low serum albumin without a corresponding proteinuria or liver dysfunction. The development of COVAKI during the hospital stay was associated with the use of glucocorticoids, hydroxychloroquines (HCQs), and heparin.

## Introduction

A third novel coronavirus leading to severe respiratory infection was identified in Wuhan, China, in December 2019 [1]. The disease had a fast worldwide spread, and WHO declared a pandemic. The clinical spectrum of COVID-19 is broad, ranging from asymptomatic to mild upper respiratory infection to critical illness. COVID-19 is a multisystem disease, and almost all the body systems are involved in varying proportions [1]. In COVID-19, viral invasion happens through the human angiotensin-converting enzyme 2 (ACE2) receptor which is expressed in various tissues including the lungs, small intestine, kidneys, heart, thyroid, testis, and adipose tissue which indicates that the virus can directly infect cells of various organ systems [2]. Initial reports across the world showed that the association of acute kidney injury (AKI) with COVID-19 was rare; however, subsequent studies from other parts of the world showed the development of AKI in 20-70% of patients with COVID-19 with higher mortality [3]. Further, various studies confirmed the 10% as the estimated incidence of AKI among all hospitalized COVID-19 patients, and of them, 4% required renal replacement therapy (RRT) [4].

In another systematic review and meta-analysis by Silver et al. in November 2020, the pooled prevalence of AKI in COVID-19 patients was 28%, and the prevalence of RRT was 9% [5]. In a study of 5,449 patients, AKI was found in 36.6 % of patients, and 14.3% of them required dialysis. Patients with AKI had higher mortality (35%) when compared to patients without AKI (16.3%) [6]. In a retrospective study of patients with COVID-19 pneumonia, those diagnosed with AKI had higher mortality (11.2%) when compared with those without AKI (1.2%) [7].

The exact mechanism of COVID-19-associated acute kidney injury (COVAKI) is unclear. Various evidence-based theories have been put forth as follows: (1) AKI results due to SARS-CoV-2 binding to the ACE2 receptors in kidney cells. Detection of coronavirus in the kidneys and urine of patients with COVID-19 supports this theory [8]. (2) In one autopsy study of kidney transplant recipients who died of COVID-19, viral inclusion structures were evident in the glomerular endothelial cells. Viral infection could induce tubular damage through the deposition of membrane attack complexes on tubules and infiltration of CD68 macrophages into the interstitium. (3) In a study of 26 autopsies of patients with COVID-19, proximal tubular cells showed loss of brush borders, vacuolar degeneration, and necrosis [9]. (4) Collapsing focal segmental glomerulosclerosis was observed in kidney biopsies of two Afro-American patients. However, SARS-CoV-2 RNA was not observed in the kidney tissue [10]. A hypothesis was put forth that a cytokine storm induced APOL1 expression on podocytes that caused immune-mediated injury. (5) Indirect mechanisms that can lead to AKI in patients with COVID-19 are infections, hemodynamic instability due to various causes, rhabdomyolysis, and renal hypoxia [11].

A few studies from India reported an association of renal injury with COVID-19. Thus, this retrospective study gave us an opportunity to study the incidence of AKI, disease trajectory with AKI, and factors associated with the occurrence of AKI in COVID-19 patients from Pune, India. The present study has been carried out with the primary objective of determining the incidence and severity of AKI in patients with SARS-CoV-2 infection. Moreover, it was aimed at determining the impact of AKI on various patient outcomes in COVID-19 and to find out the correlation between the rise of inflammatory markers and occurrence and clinical and biochemical profiles in COVID-19 patients. 

This article was previously presented as an abstract at the ISN Frontiers "Infections and the Kidneys" Meeting, New Delhi, India, on September 22-25, 2022.

## Materials and methods

This proof-of-concept single-center case-control study was carried out at Symbiosis International University (SIU) in Western Maharashtra, which was a dedicated tertiary care center during the pandemic. Four hundred and fifty-five patients with positive RT-PCR assay for SARS-CoV-2 admitted to the wards or any of the ICUs during May-June 2020 were included in this study. Approvals by the Institutional Research Committee, Independent Ethics Committee, and Data Access Review Committee were obtained before the initiation of the study. The required norms of data privacy and confidentiality were followed, and the Institutional Ethics Committee of Symbiosis International University (SIU) approved the study (approval number: SIU/IEC/221).

Inclusion criteria

We included patients above 18 years of age with positive RT-PCR reports for SARS-CoV-2 infection and admitted between May 2020 and June 2020.

Exclusion criteria

Patients below 18 years of age were excluded. Patients who were on dialysis support prior to enrolment were also excluded. We also did not include patients presenting with established AKI of known etiology.

Data collection

Data related to socio-demographic characteristics, clinical information along with the history of symptoms, comorbidity and medications, ICU requirements, oxygen saturation level, dialysis requirements, duration of ICU stay, and outcome of admission were collected.

We also collected information on past and current use of medications especially the use of immunosuppressants, RAAS blockers, metformin, and aspirin. 

Definitions of outcomes

AKI was defined as per the Kidney Disease Improving Global Outcomes (KDIGO) criteria as a change in the serum creatinine of 0.3 mg/dl over a 48-hour period or 50% increase in baseline creatinine. For patients with a previous serum creatinine in the 7-365 days prior to admission, the most recent serum creatinine value was considered the baseline creatinine. For patients without baseline creatinine in the 7-365 days prior to admission, the admission creatinine was imputed on the basis of the KDIGO AKI guidelines [12]. Hospital mortality was defined as death due to COVID-19 during hospital stay, and morbidity was estimated with length of hospital stay, length of ICU stay, requirement of dialysis, and rise of inflammatory markers. Moreover, case was defined as a patient admitted with COVID-19 (RT-PCR positive) and who developed AKI during the hospital stay, while control was defined as a patient admitted with COVID-19 (RT-PCR positive) and who did not develop AKI during the hospital stay.

Statistical analysis

We analyzed the clinical and laboratory characteristics of the 455 patients by demographic features, presence and severity of AKI, requirement of dialysis, and other morbidity parameters as a consequence of AKI. For statistical analysis, variables with skewed distributions were log-transformed to satisfy assumptions of normality. Differences in clinical and biochemical characteristics between groups of patients with AKI and without AKI were tested by ANOVA adjusting for age and sex or by chi-squared test as applicable. Analyses were carried out using R statistical programming language.

## Results

Six hundred and thirty-seven patients with confirmed SARS-CoV-2 infection required admission between May 2020 and June 2020. Of them, 455 were eligible to be included in the study as per eligibility criteria. There were 263 male and 192 female participants. Among patients who developed AKI, 28 (62.2%) were male, and 17 (37.8%) were female. As per KDIGO criteria, 34 (74.6%) had stage 1 AKI, five (11%) had stage 2 AKI, and six (13.3%) had stage 3 AKI. Patients with COVAKI were older compared to those who did not develop AKI during hospital stay (59.3 vs 47.2 y, p<0.0001).

The average age of study participants was 47.6 (+15.6) years. Of them, 130 (28.5%) had diabetes mellitus (DM), 118 (25.9%) were hypertensive, seven (1.5%) had chronic kidney disease (CKD), 15 (3.3%) had bronchial asthma, and 23 (5%) had coronary artery disease (CAD). Of 130 patients with type 2 DM, 24 (18.46%) developed AKI. Similarly, 62.2% of hypertensive patients and 15.6% of patients with CAD developed AKI (Table 1).

**Table 1:** Demographic and clinical features by gender: the table describes gender distribution among patients with COVID-19 associated with AKI. Females had a higher severity of AKI (stage 3) compared to male patients. Values are n (%), p-value by chi-squared or exact test as applicable AKI: acute kidney injury; HD: hemodialysis; SLED: sustained low-efficiency dialysis; COAD: chronic obstructive airway disease; HCQs: hydroxychloroquines; ACEi: angiotensin-converting enzyme inhibitors; ARB: angiotensin receptor blocker * Remdesivir was introduced recently and very few patients (<5%) received the antiviral

Characteristics	Male	Female	Total	P-value
AKI				
Yes	28 (10.6)	17 (8.8)	45 (9.9)	0.516
No	235 (89.4)	176 (91.2)	411 (90.1)	
Stage of AKI				
1	19 (7.2)	15 (7.8)	34 (7.5)	--
2	3 (1.1)	2 (1)	5 (1.1)	
3	0	6 (2.3)	6 (1.3)	
No AKI	235 (89.4)	176 (91.2)	411 (90.1)	
Dialysis required * (contains outlier)				
Yes	5 (1.9)	0	5 (1.1)	--
No	258 (98.1)	193 (100)	451 (78.7)	
Type of dialysis *				
HD	4 (1.5)	0	4 (0.9)	--
SLED	1 (0.4)	0	1 (0.2)	
Not required	258 (98.1)	193 (100)	451 (98.7)	
Oliguria				
Yes	5 (1.9)	0	5 (1.1)	--
No	256 (97.3)	193 (100)	449 (98.5)	
Not available	2 (0.8)	0	2 (0.4)	
Smoker				
Yes	3 (1.1)	0	3 (0.7)	--
No	260 (98.9)	193 (100)	453 (99.3)	
Obesity				
Yes	13 (5)	21 (11)	34 (7.5)	0.016
No	249 (95)	170 (89)	419 (92.5)	
Diabetes				
Yes	73 (27.8)	57 (29.5)	130 (28.5)	0.678
No	190 (72.2)	136 (70.5)	326 (71.5)	
Hypertension				
Yes	64 (24.3)	54 (28)	118 (25.9)	0.380
No	199 (75.7)	139 (72)	338 (74.1)	
Chronic kidney disease				
Yes	7 (2.7)	0	7 (1.5)	--
No	256 (97.3)	193 (100)	449 (98.5)	
Coronary artery disease				
Yes	16 (6.1)	7 (3.6)	23 (5)	0.236
No	247 (93.9)	186 (96.4)	433 (95)	
Bronchial asthma/COAD				
Yes	8 (3)	7 (3.6)	15 (3.3)	0.729
No	255 (97)	186 (96.4)	441 (96.7)	
Hypoxia				
Yes	68 (25.9)	38 (19.7)	106 (23.2)	0.124
No	195 (74.1)	155 (80.3)	350 (76.8)	
Metabolic acidosis				
Yes	11 (4.2)	4 (2.1)	15 (3.3)	0.315
No	251 (95.4)	189 (97.9)	440 (96.5)	
Not available	1 (0.4)	0	1 (0.2)	
Respiratory acidosis				
Yes	5 (1.9)	6 (3.1)	11 (2.4)	--
No	257 (97.7)	187 (96.9)	444 (97.4)	
Not available	1 (0.4)	0	1 (0.2)	
Corticosteroids				
Yes	52 (19.9)	33 (17.1)	85 (18.7)	0.446
No	209 (80.1)	160 (82.9)	369 (81.3)	
Heparin				
Yes	48 (18.4)	25 (13)	73 (16.1)	0.119
No	213 (81.6)	168 (87)	381 (83.9)	
HCQs				
Yes	45 (17.3)	30 (15.5)	75 (16.6)	0.617
No	215 (82.7)	163 (84.5)	378 (83.4)	
Remdesivir*				
Yes	0	0	0	--
No	261 (100)	193 (100)	454	
ACEi/ARB				
Yes	22 (8.4)	10 (5.2)	32 (7)	0.181
No	239 (91.6)	183 (94.8)	422 (93)	
Discharge status				
Death	9 (3.4)	3 (1.6)	12 (2.6)	0.222
Recovered	254 (96.6)	189 (98.4)	443 (97.4)	
Dialysis post-discharge				
Yes	1 (0.4)	0	1 (0.2)	--
No	253 (99.6)	188 (100)	441 (99.8)	

The average hospital stay was 14.02 days (SD=5.9) for COVID-19 patients who developed AKI, whereas it was 10.01 days (SD=3.0) for patients without AKI. During the hospital stay, 45 (9.9%) developed AKI. AKI developed in these patients was distinct from community-acquired AKI as it was not evident at the time of admission and was developed after 48 hours of hospitalization. It makes it easier to distinguish community-acquired AKI from COVAKI. Unfortunately, we could not capture data on patients arriving with AKI and differentiate clinical presentation, severity, and outcomes between community-acquired AKI and COVAKI.

We looked at levels of inflammatory biomarkers in patients developing COVAKI. We observed higher levels of CRP in males (mean 35.4 mg/dL, SD=160.5) compared to females (mean 17.0 mg/dL, SD= 25.8) with p=0.116. Likewise, ferritin was higher in males (mean 520.8 ng/ml, SD=1396.5) compared to females (mean 114.34 ng/ml, SD=152.84) with p=0.0001 being significant. Albumin being a negative inflammatory marker was low in females (mean 0.10 gm%, SD=0.53) compared to males (mean 0.23 gm%, SD=0.83) being less significant with p=0.70. We could not quantify proteinuria, and this could be a limitation in understanding the cause of low albumin in patients with COVAKI (Table 2).

**Table 2:** Biochemical characteristics of patients by gender: inflammatory markers were elevated but rise in ferritin and D-dimers was more pronounced. Albumin was significantly low in patients with AKI. Values are mean (SD), p-value by ANOVA CRP: C-reactive protein; HCO3: bicarbonate; ICU: intensive care unit; HFO2: high-flow oxygen; LFO2: low-flow oxygen

Gender	Male		Female		P-value
	Mean	Std. dev.	Mean	Std. dev.	
Age (years)	47.6	15.6	49.5	16.3	0.195
CRP (mg/dL)	35.4	160.5	17.0	25.8	0.116
Ferritin (ng/ml)	520.8	1396.5	114.3	152.8	0.0001
Albumin (gm%)	0.2	0.8	0.1	0.5	0.070
D-dimer	58.1	648.8	72.7	411.2	0.784
HCO3 (mEq/L)	22.8	5.4	26.2	5.2	0.030
Sodium (mEq/L)	135.2	6.1	137.2	3.9	0.159
Potassium (mEq/L)	4.1	0.8	3.7	0.6	0.123
Days in hospital (days)	10.2	3.4	10.3	3.9	0.327
Days in ICU (days)	1.1	3.2	1.0	2.8	0.616
Days on ventilator	0.5	2.3	0.3	1.5	0.325
HFO2 (days)	1.9	0.2	1.93	0.2	0.863
LFO2 (days)	1.7	0.4	1.8	0.3	0.109

COVAKI was associated with DM (n=24, P=0.001), hypertension (n=28, P=0.001), and CAD (n=7, P=0.01). However, chronic airway disease (p: 0.05) was not associated with the development of COVAKI. Advanced age was a risk factor for the development of AKI. The mean age of patients having COVAKI was 59.3 years and those not having AKI was 47.2 years (p<0.0001). Similarly, higher levels of D-dimer (426.3) and lower serum albumin (1.8 gm%) were associated with COVAKI (p<0.0001) (Table 3).

**Table 3:** Demographic and clinical characteristics of patients by the presence of COVAKI: patient outcomes were significantly affected by the development of COVAKI due to higher mortality and longer stay in the hospital and ICUs. Oxygen requirement was also seen higher in patients with AKI. Overall, AKI developing in patients with COVID-19 had a significant burden on the healthcare delivery system. COVAKI: COVID-19-associated AKI; HD: hemodialysis; SLED: sustained low-efficiency dialysis; COAD: chronic obstructive airway disease; HCQs: hydroxychloroquines; ACEi: angiotensin-converting enzyme inhibitor; ARB: angiotensin receptor blocker

Characteristics	Total	AKI (cases)	No AKI (controls)	P-value
Dialysis requirement *n (%)*				
Yes	5 (1.1)	5 (11.1)	0	--
No	450 (98.7)	40 (88.9)	410 (99.8)	
Type of dialysis *n (%)*				
HD	4 (0.9)	4 (8.9)	0	--
No dialysis	450 (98.7)	40 (88.9)	410 (99.8)	
SLED	1 (0.2)	1 (2.2)	0	
Oliguria *n (%)*				
Yes	5 (1.1)	5 (11.1)	0	--
No	449 (98.5)	40 (88.9)	409 (99.5)	
Not available	2 (0.4)	0	2 (0.5)	
Chronic kidney disease *n (%)*				
Yes	7 (1.5)	7 (15.6)	0	--
No	449 (98.5)	38 (84.4)	411 (100)	
Diabetes mellitus *n (%)*				
Yes	130 (28.5)	24 (53.3)	106 (25.8)	0.0001
No	326 (71.5)	21 (46.7)	305 (74.2)	
Hypertension *n (%)*				
Yes	118 (25.9)	28 (62.2)	90 (21.9)	0.0001
No	338 (74.1)	17 (37.8)	321 (78.1)	
Obesity *n (%)*				
Yes	34 (7.5)	3 (6.7)	31 (7.6)	--
No	419 (92.5)	42 (93.3)	377 (92.4)	
Smoking *n (%)*				
Yes	3 (0.7)	0	3 (0.7)	--
No	452 (99.1)	45 (100)	407 (99)	
Coronary artery disease *n (%)*				
Yes	23 (5)	7 (15.6)	16 (3.9)	0.001
No	433 (95)	38 (84.4)	395 (96.1)	
Bronchial asthma/COAD* *n (%*)				
Yes	15 (3.3)	4 (8.9)	11 (2.7)	0.050
No	441 (96.7)	41 (91.1)	400 (97.3)	
Hypoxia *n (%)*				
Yes	106 (23.2)	32 (71.1)	74 (18)	0.0001
No	350 (76.8)	13 (28.9)	337 (82)	
Metabolic acidosis *n (%)*				
Yes	15 (3.3)	12 (26.7)	3 (0.7)	0.0001
No	440 (96.5)	33 (73.3)	407 (99)	
Not available	1 (0.2)	0	1 (0.2)	
Respiratory acidosis *n (%)*				
Yes	11 (2.4)	9 (20)	2 (0.5)	--
No	444 (97.4)	36 (80)	408 (99.3)	
Not available	1 (0.2)	0	1 (0.2)	
Corticosteroid *n (%)*				
Yes	85 (18.7)	31 (68.9)	54 (13.2)	0.0001
No	369 (81.3)	14 (31.1)	355 (86.8)	
Heparin *n (%)*				
Yes	73 (16.1)	28 (62.2)	45 (11)	0.0001
No	381 (83.9)	17 (37.8)	364 (89)	
HCQs *n (%)*				
Yes	75 (16.6)	17 (38.6)	58 (14.2)	0.0001
No	378 (83.4)	27 (61.4)	351 (85.8)	
Remdesivir* *n (%)*				
Yes	0	0	0	--
No	454	45	409	
Use of ACEI/ARB *n (%)*				
Yes	32 (7)	4 (8.9)	28 (6.8)	0.544
No	422 (93)	41 (91.1)	381 (93.2)	
Discharge status *n (%)*				
Death	12 (2.6)	9 (20.5)	3 (0.7)	0.0001
Recovered	443 (97.4)	35 (79.5)	408 (99.3)	
Dialysis post-discharge *n (%)*				
Yes	1 (0.2)	1 (2.9)	0	--
No	441 (99.8)	34 (97.1)	407	

All these patients received standard treatment of care as per Indian Council of Medical Research (ICMR) guidelines. Indications to start renal replacement therapy were conventional, and we did not use any cytokine adsorbent filters in these patients. Corticosteroids were used in 85 patients; of these, 31 were patients with COVAKI (p=0.001). Heparin was used in 73 patients; among these, 28 were patients with COVAKI (p=0.0001), and 17 patients out of 75 who were on hydroxychloroquines (HCQs) had COVAKI (p=0.0001). Thus, the use of corticosteroids, heparin, and HCQs was more in COVID-19 patients with AKI compared to those patients without AKI. Among n=32 (7%) COVID-19 patients who were already on ACE or angiotensin receptor blocker (ARB), only four (8.9%) developed AKI (p=0.54).

About 106 patients had hypoxia and 32 patients (71.1%) developed AKI, which was statistically significant (p<0.0001). The average ICU stay was six days (SD=6.3) and 0.57 days (SD=2.123) for patients with and without AKI (p=0.0001), respectively. Days spent on the ventilator were 3.3 days (SD=5.3) for patients with AKI and 0.11 days (SD=0.80) for non-AKI patients (p=0.0001). There were 12 deaths (2.6%) among all COVID-19 patients. Out of these, nine (20.5%) had COVAKI. Three patients (0.7%) died who did not develop AKI in their course in the hospital (relative risk: 27.33; odds ratio: 33.92). This highlighted the statistically significant (p<0.0001) association of COVAKI with hospital mortality (Z statistics 5.115) (Table 4).

**Table 4:** Biochemical characteristics of patients with the presence of AKI. Values are mean (SD), p-value by ANOVA CRP: C-reactive protein; IL-6: interleukin 6; HCO3: bicarbonate; HFO2: high-flow oxygen; LFO2: low-flow oxygen

Gender	Patient with AKI (cases)		Patient without AKI (controls)		P-value
	Mean	SD	Mean	SD	
Age (years)	59.2	78.8	47.2	15.5	0.0001
CRP (mg/dL)	78.8	145.6	22.0	119.54	0.003
Ferritin (ng/dL)	1619.1	3030.5	114.3	152.8	0.0001
Albumin (gm%)	1.8	1.3	3.0	0.5	0.0001
IL-6 (pgm/mL)	-	-	-	-	
D-dimer (mg/L)	426.7	1577.1	24.6	255.6	0.0001
HCO3 (mEq/L)	22.8	7.3	24.7	3.09	0.200
Sodium (mEq/L)	136.6	6.3	135.1	4.3	0.307
Potassium (mEq/L)	4.2	0.9	3.7	0.5	0.027
Days in hospital	14.0	5.9	10.0	3.0	0.0001
Days in ICU	6	6.3	0.5	2.1	0.0001
Days on ventilator	3.3	5.3	0.1	0.8	0.0001
HFO2 (days)	1.6	0.4	1.9	0.1	0.0001
LFO2 (days)	1.4	0.4	1.8	0.3	0.0001

## Discussion

**Figure 1:**
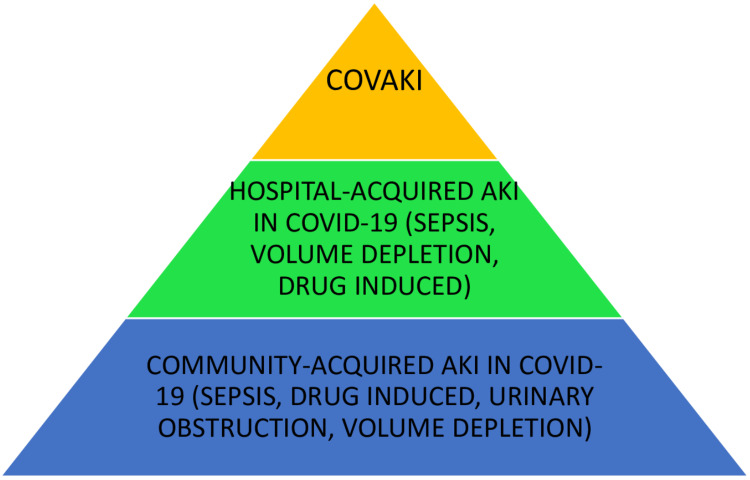
Distribution of patients with AKI: differentiating community-acquired, hospital-acquired, and COVAKI. COVAKI: COVID-19-associated acute kidney injury

This proof-of-concept case-control study assessed the impact of AKI on hospital mortality and morbidity in patients admitted with COVID-19. This was supported by estimating the risk factors for the development of COVAKI and corresponding changes in inflammatory markers in respective patients. As we excluded patients who were already on dialysis or having AKI/ESRD at arrival, new-onset AKI patients were predominantly enrolled and hence differentiated from patients with community-acquired AKI (Figure 1).

Our study was carried out during the first pandemic wave from May 2020 to June 2020. At that time, there was a lot of fear and apprehension about COVID-19, and almost all RT-PCR-positive SARS-CoV-2 patients were being hospitalized. The home quarantine concept was not much in vogue. We collected the data of 455 admitted patients, and analysis showed the incidence of AKI to be around 9.9%. So far, there have been few studies about the involvement of kidneys in COVID-19 and its impact on disease trajectory, and these showed varied results. Most of the hard outcomes were regarded as a consequence of lung involvement predominantly. We tried to focus on the fact that kidney injury was as significant as lung injury that could affect the outcomes in hospitalized patients.

Early reports from China concluded that COVID-19 did not result in significant AKI. Two cases with AKI were reported out of 645 hospitalized patients in Zhejiang from China [13].

Cheng et al. in their study of 701 patients showed that the incidence of AKI was 5.1%, whereas studies carried out in the United States showed different results [14]. Hirsch et al. in their study of 5449 admitted patients with COVID-19 demonstrated the incidence of AKI to be 36.6% [6]. Mohamed et al. found that the incidence of AKI was 28% among 575 admitted patients with COVID-19 [15]. Chan et al. reviewed data of patients from February 2020 to May 2020. Out of 3993 hospitalized patients with COVID-19, AKI was observed in 46% of patients. The proportion of patients with AKI stages 1, 2, and 3 were 39%, 19%, and 42%, respectively. In-hospital mortality was 50% in patients with AKI when compared to those without AKI (8%).

There are few Indian studies on AKI in COVID-19 patients. Sampathkumar et al. collected and analyzed the data of 718 adult patients with COVID-19 from April to November 2020 and concluded that 7% developed AKI with a mortality of 44%. These patients with AKI had raised D-dimer and low albumin levels [16]. Sindhu et al. carried out an observational study among 2650 COVID-19 patients between May 2020 and October 2020. They found the incidence of AKI at 7.2% with most of the patients having stage 1 AKI (71.1%). This study showed mortality was 22.1% in COVID-19 patients with renal injury [17]. Our study also had similar results. We found the incidence of AKI stage 1 to be 74.6%; stage 2, 11.1%; and stage 3, 13.3%. We also noticed that among the patients who succumbed, 75% had AKI.

This geographical heterogeneity in AKI incidence could be attributed to differences in hospitalization criteria, differences in healthcare systems, socioeconomic disparity, ethnicity, and the presence of comorbidity.

In our study, we found that there is a significant correlation between the occurrence of AKI with male sex, advancing age, smoking, DM, hypertension, and use of vasopressors, which is consistent with earlier studies [18]. Surprisingly, we found that earlier use of ACE inhibitors (ACEi) or ARBs had no significant influence on the occurrence of AKI in patients with COVID-19. On the contrary, the use of ACEi and ARBs appeared to be protective in COVID-19 patients. Hirsch et al. in their analytical study of COVID-19 patients with AKI had similar findings [6].

They also did not find an association of prior use of RAAS blockers on the occurrence of AKI in COVID-19 patients; similar findings were noted by Kow and Hasan [19].

On the contrary, there are studies by Lee et al. and several others that show an increased incidence of AKI in COVID-19 patients using RAAS blockers [20].

**Figure 2:**
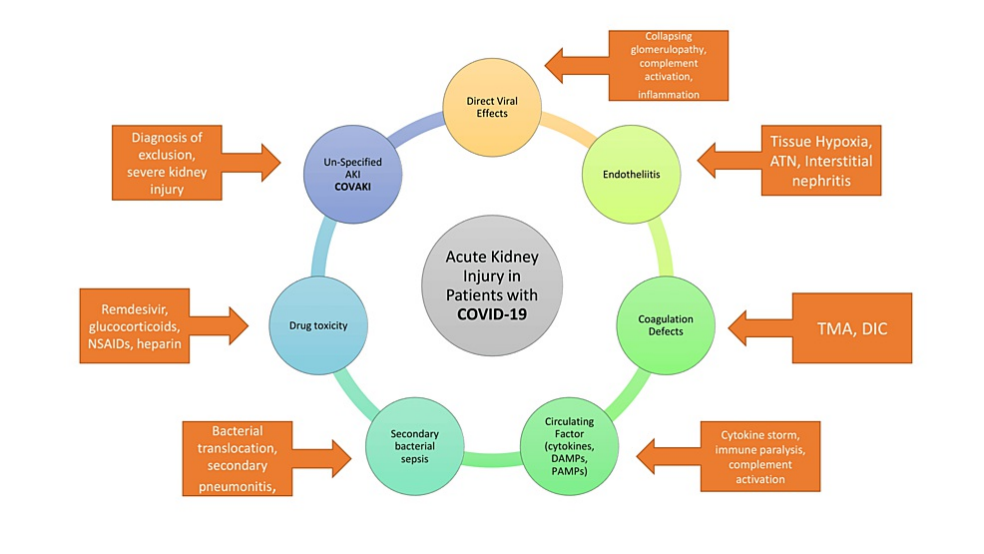
Etiopathogenesis of acute kidney injury in patients with COVID-19. ATN: acute tubular necrosis; DAMP: damage-associated nuclear pattern; PAMP: pathogen-associated nuclear pattern; NSAIDs: non-steroidal anti-inflammatory drugs; TMA; thrombotic microangiopathy; DIC: disseminated intravascular coagulation

We could identify the place of COVAKI among the various causes of injury to renal tissue. Essentially, COVAKI could be a diagnosis of exclusion after identifying all common causes of AKI in patients admitted with COVID-19 (Figure 2).

Our study was a retrospective observational study so it has its own limitations. Hospitalization criteria were different at that time. All grades of severities of sequentially admitted patients (as far as possible) were enrolled in our study. Still, we noticed that a significant proportion of patients developed AKI during the course of their disease with no identifiable etiology or diagnostic biomarker. Hence, the diagnosis of COVAKI remains a diagnosis of exclusion. We observed higher levels of inflammatory biomarkers like CRP and ferritin in patients with COVAKI during hospitalization. The role of immune activation was further strengthened by the low albumin level observed in patients with AKI. There was a statistically significant increase in the D-dimer which may suggest the role of intra-glomerular clotting in COVID-19 [21,22].

Recommendation

The phenotype of AKI, which we termed COVAKI, was difficult to differentiate from community-acquired and hospital-acquired AKI. Such sub-phenotyping of AKI might be helpful for the management and prognostication of a significant complication of AKI in patients with COVID-19. This might help us revisit the COVID-19 clinical data and access the contribution of AKI in overall mortality and morbidity vis-à-vis pulmonary injury.

Limitations

It was observed that there were differences in the presentation, complications, and outcomes in the community-acquired AKI and COVAKI. Due to the small sample size and challenges in capturing data for community-acquired AKI, further studies/data analysis is required to understand the contribution of AKI to overall mortality and morbidity during the COVID-19 pandemic.

## Conclusions

COVAKI was associated with prolonged hospital stay, days on ventilator, and higher oxygen requirement and consequently higher morbidity and mortality. Risk factors for developing COVAKI were associated with older age, DM, hypertension, CAD, metabolic acidosis, and hypoxia. Inflammatory markers were increased in COVAKI compared to those without kidney injury, but the causal role could not be demonstrated. The development of COVAKI during the hospital stay was associated with the use of glucocorticoids, HCQs, and heparin. ACEi and ARB use was not associated with a higher incidence of COVAKI.
